# A Systematic Review With Targeted Meta-Analyses of Curcumin and Berberine In Vitro Cytotoxicity Models (2014-2026)

**DOI:** 10.7759/cureus.104165

**Published:** 2026-02-24

**Authors:** Suresh K, Saravanasingh Karan Chand Mohan Singh, Shalini Boopathi, Sugumaran P Pichamuthu, Nithyamala I, Gayatri R

**Affiliations:** 1 Department of Pediatrics (Kuzhanthai Maruthuvam), National Institute of Siddha, Chennai, IND; 2 Department of Medicine (Maruthuvam), National Institute of Siddha, Chennai, IND; 3 Department of Special Medicine, Government Siddha Medical College, Chennai, IND; 4 Department of Pharmacology (Gunapadam), National Institute of Siddha, Chennai, IND; 5 Department of Pathology (Noinaadal), National Institute of Siddha, Chennai, IND

**Keywords:** berberine, curcumin, cytotoxicity, meta-analysis, systematic review

## Abstract

Traditional systems of medicine (siddha, ayurveda, and traditional Chinese medicine (TCM)) contribute a large fraction of natural products investigated for anticancer activity. Yet quantitative synthesis is often invalidated by extreme methodological heterogeneity (different cell lines, exposure times, and viability assays). Therefore, we performed a Preferred Reporting Items for Systematic Reviews and Meta-Analyses (PRISMA)-guided systematic review of preclinical studies between 2014 and 2026, and conducted targeted meta-analyses only in narrowly matched strata where pooling is defensible, i.e., (i) curcumin against Michigan Cancer Foundation-7 (MCF-7) breast cancer cells at 48 hours using MTT-like viability assays and (ii) berberine against HepG2 hepatocellular carcinoma cells at 48 hours using MTT-like assays. Effect sizes were half-maximal inhibitory concentration (IC50) values, analyzed on the log scale with random-effects (restricted maximum likelihood (REML)) models. When publications did not report variance for IC50, a conservative within-study coefficient of variation of 20% was assumed and explored in sensitivity analyses.

Across three curcumin studies (MCF-7, 48 hours), the pooled geometric mean IC50 was 22.85 µM (95% CI: 11.04-47.27) with high heterogeneity (I² = 93.54%). Across two berberine studies (HepG2, 48 hours), the pooled geometric mean IC50 was 31.63 µM (95% CI: 22.84-43.79) with moderate heterogeneity (I² = 63.70%). Mechanistically, the included studies converge on apoptosis induction and anti-metastatic signaling: curcumin downregulated anti-apoptotic nodes (e.g., Mcl-1) and modulated microRNAs, while berberine affected epithelial-mesenchymal transition (EMT)-linked pathways (e.g., transforming growth factor-beta (TGF-β)/Smad), telomerase-associated phenotypes, and metabolic transport targets. We additionally map representative siddha herbo-mineral and ayurvedic polyherbal preclinical evidence and discuss translational obstacles (standardization, bioavailability, and reporting quality). To improve preclinical data transparency, we recommend protocol preregistration, such as Clinical Trials Registry - India (CTRI), and public deposition of extracted datasets and analysis scripts.

## Introduction and background

Traditional Asian medical systems, particularly siddha, ayurveda, and traditional Chinese medicine (TCM), have long used botanical, mineral, and herbo-mineral preparations for chronic disease management, including conditions now recognized as malignant diseases. These systems remain scientifically relevant because natural products have historically contributed disproportionately to anticancer drug discovery, and modern integrative oncology increasingly examines traditional therapies as potential sources of bioactive compounds and mechanistic leads [1]. Despite the rapid growth of the preclinical evidence base, interpretation remains difficult because studies often differ substantially in experimental design, including model systems, exposure durations, assay chemistries, and reporting completeness [2,3]. This heterogeneity limits cross-study comparability and frequently undermines broad quantitative synthesis.

Two compounds commonly studied across these traditions are curcumin and berberine, both frequently evaluated in in vitro cytotoxicity models and therefore serving as pragmatic candidates for targeted pooling under tightly matched experimental conditions [4-9]. Several additional challenges constrain translation. For curcumin, poor systemic bioavailability and chemical instability are major barriers, motivating delivery and formulation strategies [10,11]. Berberine similarly exhibits low oral bioavailability and complex pharmacokinetics that complicate extrapolation from in vitro potency to clinically achievable exposures [12]. Moreover, complex siddha and ayurvedic herbo-mineral and polyherbal formulations introduce additional variability through differences in sourcing, processing, and standardization; recent reports underscore the need for rigorous chemical characterization and reproducible reporting in such studies [13,14]. The aim of this review is twofold: (1) to systematically identify and narratively synthesize preclinical evidence (2014-2026) regarding anticancer activity from siddha, ayurveda, TCM, and related traditional systems; and (2) to conduct targeted meta-analyses restricted to narrowly comparable experimental strata defined by cell line, exposure duration, and MTT-like viability assays to generate defensible quantitative estimates of in vitro cytotoxic potency for curcumin and berberine [2-9].

## Review

Methodology

**Figure 1:**
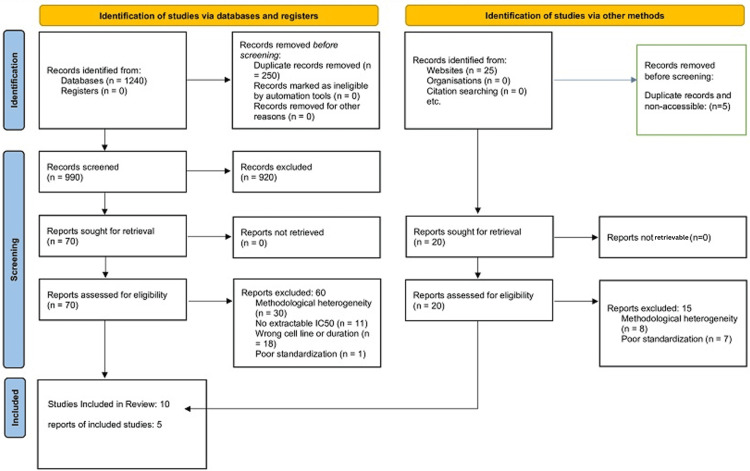
The PRISMA 2020 flow diagram The diagram outlines study identification, screening, eligibility assessment, and inclusion of studies published between 2014 and 2026 for the targeted meta-analyses and systematic review. PRISMA: Preferred Reporting Items for Systematic Reviews and Meta-Analyses [2], IC50: Half-maximal inhibitory concentration

This systematic review was prepared in accordance with the Preferred Reporting Items for Systematic Reviews and Meta-Analyses (PRISMA) 2020 statement and the PRISMA extension for search reporting (PRISMA-S) [2,3]. Because preclinical systematic reviews are not routinely eligible for clinical trial registries, we adopted a transparency approach analogous to the Clinical Trials Registry-India (CTRI). We preregistered the protocol and analysis plan in an open platform (Open Science Framework; Center for Open Science, Charlottesville, VA, USA); shared extracted data tables (half-maximal inhibitory concentration (IC50) values, assay conditions, and model metadata) and analysis scripts; and reported protocol deviations explicitly. Study identification, screening, eligibility assessment, and inclusion are summarized in the PRISMA flow diagram shown in Figure 1.

Eligibility Criteria

We included 15 preclinical studies published between January 2014 and January 2026 that evaluated the anticancer activity of siddha, ayurveda, TCM, or other traditional systems using either in vitro cancer cell line models with viability or proliferation endpoints and/or in vivo rodent tumor models with tumor burden or survival endpoints. For quantitative synthesis, studies were additionally required to match a prespecified pooling stratum (cell line, 48-hour exposure, and MTT-like viability assay) and to report an extractable IC50 value for the compound of interest.

Information Sources and Search Strategy

Searches were conducted on PubMed and PubMed Central and supplemented with structured web searches to locate full texts, using combinations of controlled vocabulary and the following keywords: system of medicine (siddha OR ayurveda OR “traditional chinese medicine” OR TCM), compound or formulation (curcumin, berberine, rasathailam, vaalai rasa mezhugu, rasagandhi mezhugu, V2S2), and model and endpoint (MCF-7, HepG2, IC50, MTT, cytotoxicity, apoptosis). Reference lists of eligible articles and relevant reviews were screened for additional studies [2,3].

Study Selection and Data Extraction

Two independent reviewers screened titles and abstracts, followed by full texts against eligibility criteria. Conflicts (include/exclude/unclear) were resolved by discussion and consensus. From each included study, we extracted the model (cell line or animal strain), exposure duration, assay type, IC50 (and variance if reported), and mechanistic endpoints (e.g., caspase activation, B-cell lymphoma 2 (Bcl-2) family changes, epithelial-mesenchymal transition (EMT) markers, microRNA (miRNA) changes). When IC50 values were only available in figures, values were extracted when numerically stated in figure labels or captions. Study selection is summarized in Figure 1. Targeted quantitative synthesis was feasible for two tightly matched strata: curcumin in Michigan Cancer Foundation-7 (MCF-7) breast cancer cells at 48 hours with MTT-like assays (three studies) and berberine in HepG2 hepatocellular carcinoma cells at 48 hours with MTT-like assays (two studies) [4-8]. 

Risk of Bias (RoB) and Reporting Quality

For in vivo studies, reporting quality was assessed using the Animal Research: Reporting of In Vivo Experiments (ARRIVE) 2.0 guidance, and RoB was considered using the Systematic Review Centre for Laboratory Animal Experimentation (SYRCLE) risk-of-bias tool where applicable (Table 1) [15,16]. For in vitro studies, key reproducibility variables (cell line authentication, mycoplasma testing, solvent controls, and replicate structure) were recorded (Table 2), recognizing that validated RoB tools for in vitro cytotoxicity are not standardized. The summarized data obtained from the 15 included studies are shown in Table 3. 

**Table 1:** In vivo RoB results (SYRCLE domains) SYRCLE: Systematic Review Centre for Laboratory Animal Experimentation [16], RoB: Risk of bias, DAL: Dalton’s ascitic lymphoma

Study	Model	Intervention	Sequence generation (low/high/unclear)	Baseline characteristics (low/high/unclear)	Allocation concealment (low/high/unclear)	Random housing (low/high/unclear)	Blinding (performance; low/high/unclear)	Random outcome assessment (low/high/unclear)	Blinding (detection; low/high/unclear)	Incomplete outcome data (low/high/unclear)	Selective outcome reporting (low/high/unclear)	Other bias (low/high/unclear)	Overall SYRCLE RoB (low/some concerns/high)
Augustine et al., 2014 [17]	Swiss albino mice (Dalton’s ascitic lymphoma (DAL) model)	Leucas aspera	Unclear	Unclear	Unclear	Unclear	Unclear	Unclear	Unclear	Unclear	Unclear	Unclear	Some concerns

**Table 2:** In vitro reproducibility/RoB results (study-level) RoB: Risk of bias, MCF-7: Michigan Cancer Foundation-7, HeLa: Henrietta Lacks, SCC: Squamous-cell carcinoma, NSCLC: Non-small cell lung cancer

Study	Model type	Cell line/model	Intervention	Cell line authentication reported (yes/no/not recorded (NR))	Mycoplasma testing reported (yes/no/NR)	Solvent/vehicle controls reported (yes/no/NR)	Replicate structure reported (biological/technical/NR)	Selective outcome reporting concerns (low/unclear/high)	Overall RoB /reproducibility judgment (low/some concerns/high)
Koohpar et al., 2015 [4]	In vitro	MCF-7	Curcumin	NR	NR	NR	NR	Unclear	Some concerns
Passos et al., 2023 [5]	In vitro	MCF-7	Curcumin	NR	NR	NR	NR	Unclear	Some concerns
Rezaeidian et al., 2024 [6]	In vitro	MCF-7	Curcumin	NR	NR	NR	NR	Unclear	Some concerns
Du et al., 2021 [7]	In vitro	HepG2	Berberine	NR	NR	NR	NR	Unclear	Some concerns
Noureini & Wink, 2015 [8]	In vitro	HepG2	Berberine	NR	NR	NR	NR	Unclear	Some concerns
Yu et al., 2014 [9]	In vitro	HepG2	Berberine	NR	NR	NR	NR	Unclear	Some concerns
Jeevanath et al., 2024 [13]	In vitro	Henrietta Lacks (HeLa)	*Thanga uram* (siddha metallo-mineral)	NR	NR	NR	NR	Unclear	Some concerns
Girija et al., 2024 [14]	In vitro	HeLa	*Nandhi mezhugu* (siddha)	NR	NR	NR	NR	Unclear	Some concerns
Li et al., 2017 [18]	In vitro	HepG2	Berberine	NR	NR	NR	NR	Unclear	Some concerns
Charak et al., 2021 [19]	In vitro	Squamous-cell carcinoma (SCC)-40	V2S2 (ayurvedic polyherbal)	NR	NR	NR	NR	Unclear	Some concerns
Pathiranage et al., 2020 [20]	In vitro	Non-small cell lung cancer (NSCLC) cells	Traditional polyherbal mixture	NR	NR	NR	NR	Unclear	Some concerns

**Table 3:** Summary of IC50 findings and mechanisms for curcumin, berberine, and related compounds in cancer cell lines (MCF-7, HepG2, HeLa, SCC-40, etc.) EMT: Epithelial-mesenchymal transition, TCM: Traditional Chinese medicine, MCF-7: Michigan Cancer Foundation-7, SCC: Squamous-cell carcinoma, DAL: Dalton's ascitic lymphoma, TGF-β: Transforming growth factor beta, AMPK: Adenosine 5′-monophosphate-activated protein kinase, PI: Propidium iodide, NF-κB: Nuclear factor kappa-light-chain-enhancer of activated B cells, HeLa: Henrietta Lacks, SCC: Squamous-cell carcinoma, NSCLC: Non-small cell lung cancer

Authors and year of publication	Model	Intervention/Medicine	Key findings/IC50 data
Koohpar et al., 2015 [4]	MCF-7 (breast cancer)	Curcumin	IC50: 53.18 µM (48h, MTT); mechanism: downregulation of Mcl-1 gene expression; induction of apoptosis
Passos et al., 2023 [5]	MCF-7 (breast cancer)	Curcumin	IC50: 11.21 µM (48h, MTT-like); mechanism: cell cycle arrest and apoptosis; assessed co-treatment with melphalan
Rezaeidian et al., 2024 [6]	MCF-7 (breast cancer)	Curcumin	IC50: 20.00 µM (48h, MTT); mechanism: apoptosis induction via miR-15a alteration
Du et al., 2021 [7]	HepG2 (liver cancer)	Berberine	IC50: 25.01 µM (48h, MTT-like); mechanism: suppression of EMT via transforming growth factor beta (TGF-β)/Smad pathway
Noureini & Wink, 2015 [8]	HepG2 (liver cancer)	Berberine	IC50: 39.99 µM (converted from 14.87 µg/mL; 48h, MTT); mechanism: telomerase inhibition and apoptosis induction
Yu et al., 2014 [9]	HepG2 (liver cancer)	Berberine	Mechanism: Apoptotic and autophagic death requiring adenosine 5′-monophosphate-activated protein kinase (AMPK) activation
Jeevanath et al., 2024 [13]	HeLa (cervical cancer)	Bisulphurette of tin (siddha metallo-mineral)	Mechanism: Dose-dependent cytotoxicity with apoptotic signatures (annexin V/propidium iodide (PI) staining)
Girija et al., 2024 [14]	HeLa (cervical cancer)	Nandhi wax (siddha)	Mechanism: In vitro cytotoxic, antiproliferative, and apoptotic activity
Li et al., 2017 [18]	HepG2 (liver cancer)	Berberine	Mechanism: Induction of apoptosis via downregulation of nuclear factor kappa-light-chain-enhancer of activated B cells (NF-κB) signaling
Charak et al., 2021 [19]	SCC-40 (oral carcinoma)	V2S2 (ayurvedic polyherbal)	Mechanism: In vitro growth inhibition and evaluation of anticancer activity
Pathiranage et al., 2020 [20]	NSCLC	Traditional polyherbal mixture	Mechanism: Evaluation of anticancer effects of a pharmaceutically viable extract
Augustine et al., 2014 [17]	DAL (mice)	Leucas aspera	Mechanism: Inhibition of tumor growth in Swiss albino mice (in vivo model)
Prasad et al., 2014 [10]	Review	Curcumin	Limited curcumin bioavailability
Hegde, et al. 2023 [11]	Review	Curcumin	Safe and well-tolerated at high doses (2 g to 12 g per day) over six to 12 months
Ai et al., 2021 [12]	Review	Berberine derived from *Rhizoma coptidis* (TCM)	Berberine against vascular diseases shows promise

Statistical Analysis

The IC50 values were analyzed on the natural logarithmic scale to reflect their approximate log-normal distribution. Random-effects meta-analyses were performed using restricted maximum likelihood (REML) estimation of the between-study variance (tau-squared (τ²)). Results are reported as pooled geometric mean IC50 values with 95% CI and 95% prediction intervals, back-transformed to micromolar (µM). Heterogeneity was summarized using the I-squared statistic (I²). When studies did not report uncertainty for IC50, a conservative within-study coefficient of variation of 20% was assumed (sensitivity range: 10% to 30%). Statistical analyses were performed using R, version 4.3.1 (R Foundation for Statistical Computing, Vienna, AUT). Meta-analyses were conducted using the Metafor package, version 4.2-0 (Wolfgang Viechtbauer, Maastricht, NLD).

Results

Targeted Meta-Analysis of Curcumin MCF-7 48-Hour MTT-Like Assays

**Figure 2:**
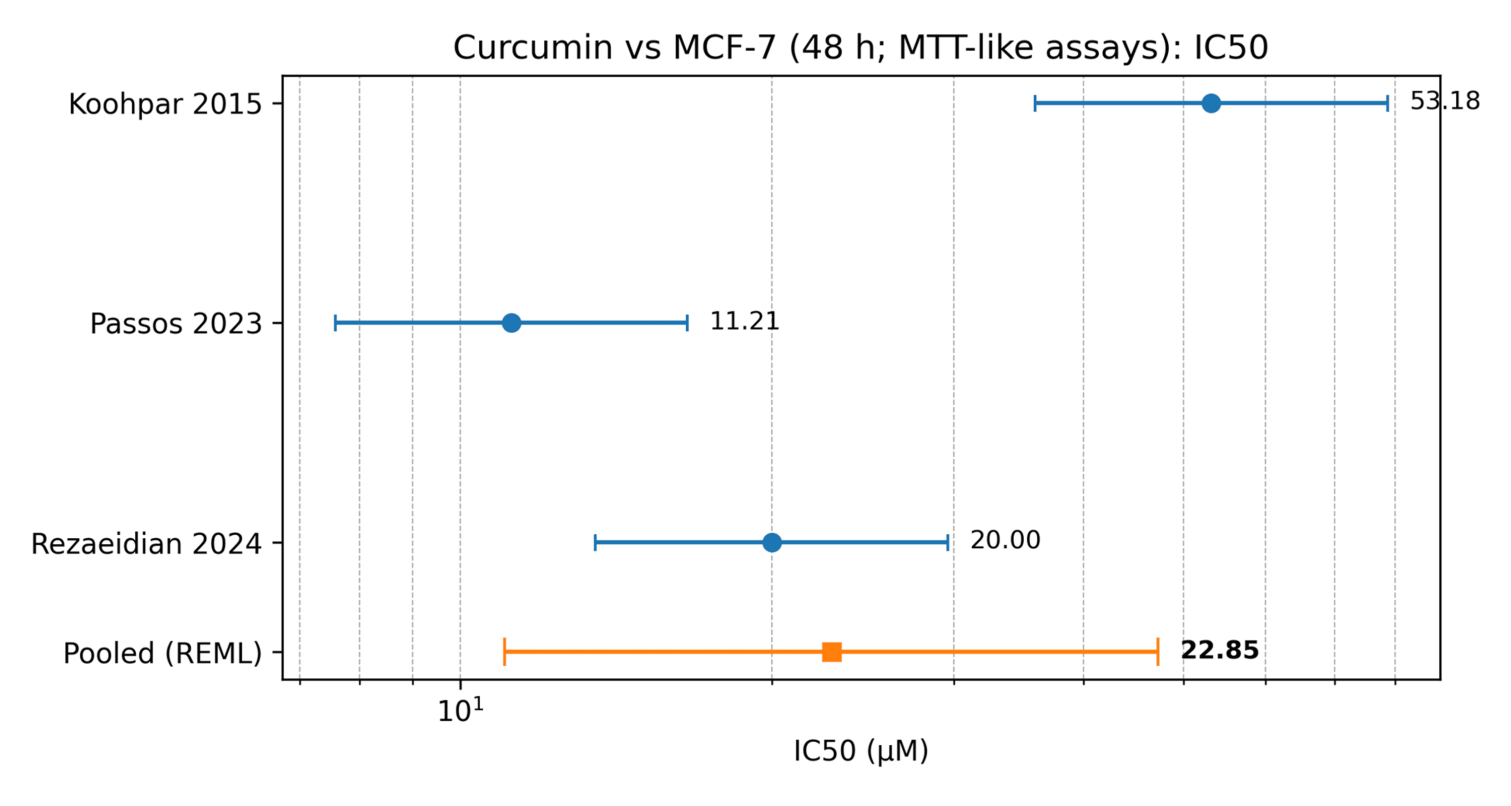
Forest plot of curcumin IC50 values in MCF-7 cells at 48 hours (MTT-like assays) The IC50 values for curcumin in MCF-7 cells at 48 hours using MTT-like viability assays are sourced from three included studies [4-6]. MCF-7: Michigan Cancer Foundation-7, IC50: Half-maximal inhibitory concentration

Three studies [4-6] reported IC50 values for curcumin in MCF-7 cells at 48 hours using MTT-like viability assays. The IC50 values ranged from 11.21 to 53.18 µM. The pooled geometric mean IC50 was 22.85 µM (95% CI: 11.04-47.27). Between-study heterogeneity was high (I² = 93.54%), with a wide 95% prediction interval (5.63-92.67 µM), indicating that experimental context and/or curcumin preparation likely modify observed potency. The curcumin-targeted meta-analysis is shown in Table 4. A forest plot of curcumin IC50 values in MCF-7 cells at 48 hours is shown in Figure 2. Representative mechanistic evidence includes berberine-mediated NF-kappaB signaling [18]. 

**Table 4:** Included studies for the curcumin targeted meta-analysis IC50: Half-maximal inhibitory concentration

Study	Model	Assay/time	IC50	Mechanistic endpoints	Notes
Koohpar et al., 2015 [4]	MCF-7	MTT; 48 hours	53.18 µM	Mcl-1 gene expression	IC50 reported in the figure label
Passos et al., 2023 [5]	MCF-7	MTT-like; 48 hours	11.21 µM	Proliferation/invasion; epigenetic markers	Co-treatment context reported; curcumin-alone IC50 extracted
Rezaeidian et al., 2024 [6]	MCF-7	MTT; 48 hours	20.0 µM	Apoptosis markers; miR-15a	IC50 stated in text

Targeted Meta-Analysis of Berberine in HepG2 48-Hour MTT-Like Assays

**Figure 3:**
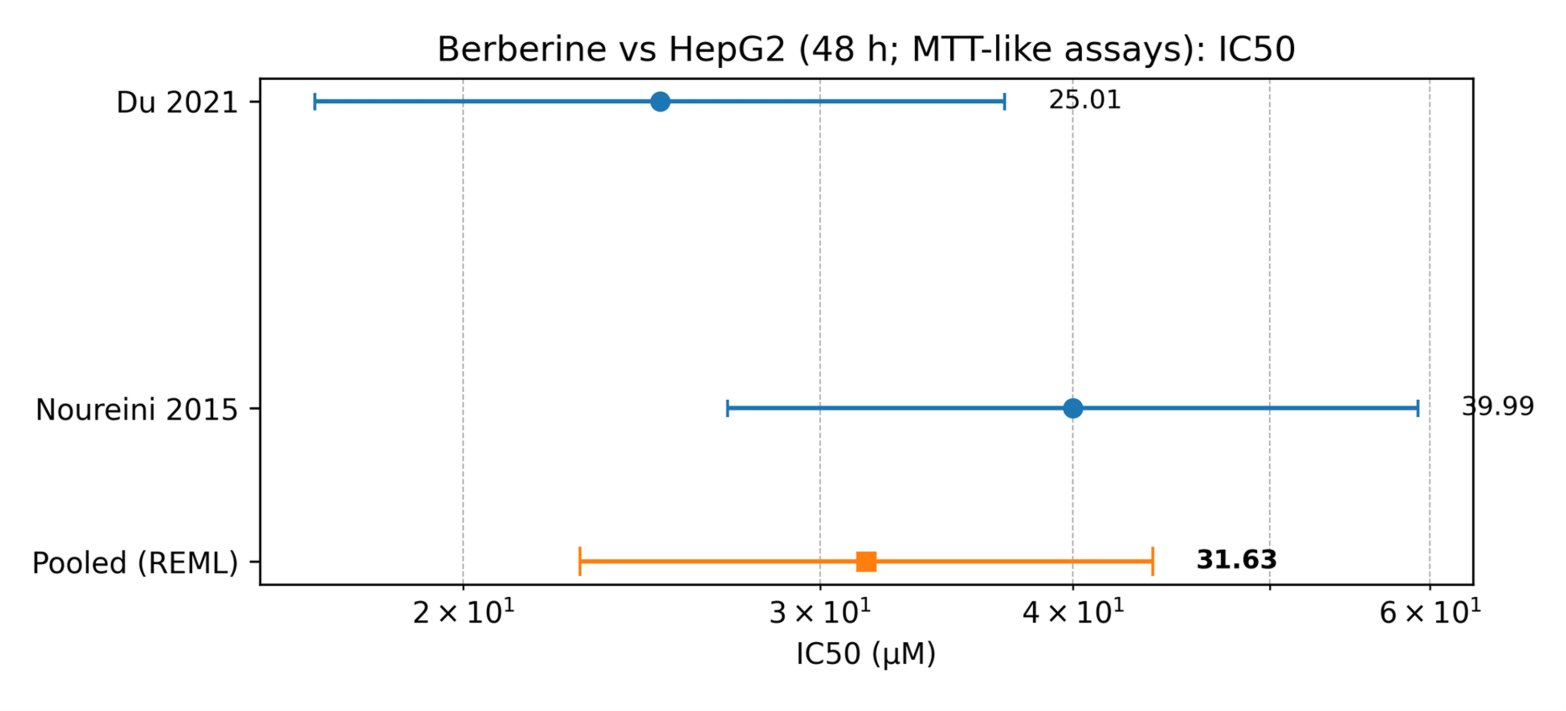
Forest plot of berberine IC50 values in HepG2 cells at 48 hours (MTT-like assays) The berberine IC50 values in HepG2 cells at 48 hours (MTT-like assays) are sourced from two included studies [7,8]. REML: Restricted maximum likelihood, IC50: Half-maximal inhibitory concentration

Two studies [7,8] reported IC50 values for berberine in HepG2 cells at 48 hours using MTT-like assays and reported results in molar concentration or convertible units. The IC50 values were 25.01 µM and 14.87 µg/mL (converted to 39.99 µM assuming berberine chloride, MW 371.8 g/mol). The pooled geometric mean IC50 was 31.63 µM (95% CI: 22.84-43.79), with moderate heterogeneity (I² = 63.70%). The berberine-targeted meta-analysis is shown in Table 5. The forest plot of berberine IC50 values in HepG2 cells at 48 hours is shown in Figure 3.

**Table 5:** Included studies for the berberine targeted meta-analysis EMT: Epithelial-mesenchymal transition, IC50: Half-maximal inhibitory concentration

Study	Model	Assay/time	IC50	Mechanistic endpoints	Notes
Du et al., 2021 [7]	HepG2	MTT-like; 48 hours	25.01 µM	EMT markers; migration/invasion	IC50 reported with high precision in the manuscript
Noureini & Wink, 2015 [8]	HepG2	MTT; 48 hours	39.99 µM	Telomerase-linked phenotypes; apoptosis	Converted from 14.87 µg/mL

Evidence Map Across Traditional Systems

Beyond single-compound exemplars, the broader preclinical literature (Table 6) includes siddha herbo-mineral formulations, ayurvedic polyherbal extracts, and TCM herbs and formulae. However, formulation complexity and variable standardization complicate cross-study comparability. Representative examples identified in this review are summarized in Table 7.

**Table 6:** Certainty/strength of evidence for preclinical outcomes IC50: Half-maximal inhibitory concentration, MCF-7: Michigan Cancer Foundation-7, RoB: Risk of bias, REML: Restricted maximum likelihood, NR: Not recorded, DAL: Dalton’s ascitic lymphoma, SYRCLE: Systematic Review Centre for Laboratory Animal Experimentation [16], ARRIVE: Animal Research: Reporting of In Vivo Experiments [15], SCC: Squamous-cell carcinoma, NSCLC: Non-small cell lung cancer

Outcome/evidence stratum	Model type	Population/model	Intervention vs. control	No. of studies (k)	Total units	Effect estimate (summary)	RoB (downgrade?)	Inconsistency (downgrade?)	Indirectness (downgrade?)	Imprecision (downgrade?)	Publication/reporting bias (downgrade?)	Other considerations (upgrade/downgrade?)	Overall certainty (high/moderate/low/very low)	Comments/rationale
IC50 (log scale) at 48 hours	In vitro	MCF-7 breast cancer cell line	Curcumin vs. vehicle	3	NR	Random-effects pooled estimate (REML) on log (IC50)	To complete (use RoB table)	To complete (I²/τ²; downgrade if substantial)	To complete (in vitro → clinical)	Likely downgrade (small k; wide CI)	Not assessed (k<10) — state feasibility	Assay/conditions tightly matched (strength within stratum)	To complete	Suggested starting point: Low (small k), adjust after RoB results
IC50 (log scale) at 48 hours	In vitro	HepG2 liver cancer cell line	Berberine vs vehicle	2	NR	Random-effects pooled estimate (REML) on log (IC50)	To complete (use RoB table)	Not estimable / very limited (k=2)	To complete (in vitro → clinical)	Likely downgrade (very small k)	Not assessed (k<10) — state feasibility	Assay/conditions tightly matched (strength within stratum)	To complete	Suggested starting point: Very low (k=2), adjust after RoB results
Anti-tumor activity endpoints (narrative evidence map)	In vivo (preclinical)	Mouse DAL model	Extract/formulation vs. control	1	NR	Narrative synthesis (no pooling)	To complete (SYRCLE)	NA (no pooling)	Potential downgrade (animal model → humans)	Likely downgrade (single study)	Not assessed — discuss qualitatively	Dose relevance, controls, blinding, outcome definition	To complete	Suggested starting point: Very low (single study), adjust after SYRCLE/ARRIVE
Cytotoxicity/mechanistic endpoints (narrative evidence map)	In vitro	Various cell lines (e.g., HeLa, SCC-40, NSCLC)	Polyherbal/siddha/ayurveda formulations vs. control	NR	NR	Narrative synthesis (no pooling)	To complete (reproducibility checklist)	NA (heterogeneous)	Downgrade (heterogeneous interventions/models)	Downgrade (variable reporting; no common effect)	Not assessed — discuss qualitatively	Formulation standardization, extraction, comparators	To complete	Suggested starting point: Very low (heterogeneous, non-pooled), adjust after extraction

**Table 7:** Representative preclinical evidence examples by traditional system TCM: Traditional Chinese medicine, EMT: Epithelial-mesenchymal transition, MCF-7: Michigan Cancer Foundation-7, SCC: Squamous-cell carcinoma, IC50: Half-maximal inhibitory concentration

System	Example medicine	Model	Reported endpoints	Key reported finding
Siddha	*Thanga uram *(siddha metallo-mineral) [13]	HeLa cervical cancer cells	MTT; annexin V/propidium iodide (PI)	Dose-dependent cytotoxicity with apoptotic signatures
Ayurveda	Polyherbal formulation V2S2 [19]	SCC-40 oral carcinoma cells	Viability/cytotoxicity	In vitro growth inhibition reported in oral carcinoma model
Ayurveda	Curcumin [4-6]	MCF-7 breast cancer cells	MTT; IC50; apoptosis markers	Pooled IC50 22.85 µM at 48 hours (high heterogeneity)
TCM	Berberine [7,8,9]	HepG2 hepatocellular carcinoma cells	MTT; IC50; EMT/telomerase-linked markers	Pooled IC50 31.63 µM at 48 hours

Discussion

This review applied a deliberately restrictive quantitative synthesis strategy to address a core obstacle in preclinical systematic reviews, i.e., extreme methodological heterogeneity [2,3]. When pooling was constrained to closely comparable experimental strata, both curcumin and berberine demonstrated reproducible in vitro cytotoxicity in widely used cancer models, with pooled IC50 values in the tens of micromolar range at 48 hours. For curcumin, the pooled geometric mean IC50 was 22.85 µM but with very high heterogeneity (I² = 93.54%) [4-6]. This level of heterogeneity suggests that curcumin preparation, assay execution, and experimental context likely influence observed potency. Mechanistic endpoints within the included studies implicate apoptosis-related pathways and regulation of anti-apoptotic signaling, including altered myeloid cell leukemia 1 (Mcl-1) expression and miRNA-associated effects [4,6]. For berberine, the pooled geometric mean IC50 was 31.63 µM with moderate heterogeneity (I² = 63.70%) [7,8]. Reported mechanisms commonly implicated EMT-linked signaling, migration, and invasion phenotypes, and TGF-β/Smad pathway involvement [7,8]. Additional studies outside the strict pooling stratum provide a supportive mechanistic context, including NF-κB-linked apoptosis signaling and AMPK-associated cell death signaling in HepG2 models [9,17].

Across traditional systems, evidence from siddha and ayurveda includes complex herbo-mineral and polyherbal formulations with reported cytotoxic and apoptotic signatures in vitro; however, reproducibility depends heavily on chemical characterization and standardization, particularly when mineral-containing preparations are used [13,14]. Ayurvedic polyherbal preparations have been evaluated in carcinoma cell models [19], and pharmaceutically viable polyherbal mixtures have been studied in NSCLC settings [20]. In vivo evidence exists in related traditional medicine investigations, such as DAL models, but integration with in vitro potency estimates requires careful interpretation due to pharmacokinetic and exposure constraints [17].

Bioavailability remains a major bottleneck for curcumin, and delivery strategies are central to any translational argument [10,11]. Berberine’s pharmacokinetics similarly complicate translation from in vitro potency to clinically achievable tissue concentrations [12]. These realities reinforce the importance of aligning preclinical experimental exposures with physiologically plausible ranges, implementing chemical fingerprinting (e.g., high-performance liquid chromatography (HPLC)), and reporting formulation details rigorously.

Limitations

This review has several limitations. First, the targeted meta-analyses were restricted to small numbers of studies due to strict matching criteria; results should therefore be interpreted as hypothesis-generating rather than definitive. Second, because many IC50 reports did not provide variance estimates, a within-study coefficient of variation was assumed for meta-analytic weighting. Although sensitivity analyses did not materially change pooled point estimates, confidence intervals depend on this assumption. Third, the evidence mapping across siddha and some polyherbal formulations included heterogeneous sources with variable reporting quality, underscoring the need for standardized reporting and chemical characterization rather than serving as strong confirmation of efficacy [13-16].

## Conclusions

When quantitative synthesis is restricted to carefully matched experimental conditions, curcumin and berberine demonstrate consistent in vitro anticancer activity in widely used models (MCF-7 and HepG2), with pooled IC50 values in the tens of micromolar range at 48 hours. Nevertheless, methodological heterogeneity, incomplete reporting, and translational constraints, particularly bioavailability and standardization, remain major barriers to robust synthesis and clinical inference. Future preclinical investigations should adopt standardized reporting practices, comprehensive chemical characterization, and transparent data sharing to enable credible evidence synthesis and to support rational development within integrative oncology.
